# Effects of variable incubation temperature during embryonic days 10–13 on leg muscle transcriptomics in day-old broiler and layer chicks

**DOI:** 10.1038/s41598-026-67770-w

**Published:** 2026-08-28

**Authors:** Umra Rasool, Henry Reyer, Michael Oster, Nares Trakooljul, Ina Starke, Carsten Krischek, Siriluck Ponsuksili, Klaus Wimmers

**Affiliations:** 1 Research Institute for Farm Animal Biology (FBN), Wilhelm-Stahl-Allee 2, 18196 Dummerstorf, Germany; 2 Institute of Food Quality and Food Safety, University of Veterinary Medicine Hannover, Bünteweg 2, Hannover, Germany; 3 Faculty of Agriculture, Civil and Environmental Engineering, University of Rostock, Justus-von-Liebig-Weg 6, 18059 Rostock, Germany

**Keywords:** Avian embryogenesis, Developmental plasticity, Hatch, Myogenesis, RNA sequencing, Skeletal muscle, Cell biology, Developmental biology, Genetics, Molecular biology, Physiology, Zoology

## Abstract

In chickens, secondary myogenesis occurs during embryonic days (ED) 10–13, which is a critical developmental window sensitive to incubation temperature as a key environmental factor. This study investigates transcriptional profiles in leg muscle of broiler (Ross 308; Ross) and layer chickens (Lohmann Brown; LB) at hatch following transient ± 1 °C deviations from standard incubation temperature during ED 10–13. Eggs were incubated at 37.8 °C (CON), 36.8 °C (Low), or 38.8 °C (High) for 72 h between ED 10–13 before being returned to standard conditions until hatch (ED 21). Leg muscle samples from both sexes were analysed using RNA sequencing (*n* = 108). Hatch body weight and yolk-free body weight were higher in Ross compared to LB, but were unaffected by incubation temperature. Lowered incubation temperature in LB hatchlings was associated with altered mitochondrial and metabolic pathways, including the downregulation of genes involved in oxidative phosphorylation and reduced expression of myogenic regulators such as *MYF5* and *MYOG* compared to CON birds. Increased incubation temperature involved enrichment of proteolytic processes and downregulation of genes associated with extracellular matrix organization (*COL1A1*, *COL6A1*) in LB hatchlings and myogenesis (*MYOG*) in Ross hatchlings compared to CON birds. Results suggest that deviations from standard incubation temperature during the critical window of secondary myogenesis modulate leg muscle development in a strain-specific manner.

**Supplementary Information:**

The online version contains supplementary material available at https://doi.org/10.1038/s41598-026-67770-w.

## Introduction

Chick embryonic development is influenced by multiple incubation conditions, including humidity, light exposure, gas exchange, egg rotation rate, and temperature, with implications on post-hatching viability and performance^1,2^. Optimal hatchability in chickens is achieved at egg incubation temperatures between 37.5 and 37.8 °C, as reported previously for both layer and broiler lines^3^.

Chicken skeletal muscle development occurs in two distinct phases during embryonic myogenesis. During the primary phase at embryonic days (ED) 4–7, embryonic myoblasts fuse to form primary myofibres, which include both fast- and slow-twitch fibres and serve as a structural scaffold for subsequent muscle development^4,5^. In the secondary phase, which extends from approximately ED 8 to ED 15, foetal myoblasts fuse around these primary myofibres to form secondary muscle fibres. This phase also gives rise to adult myoblasts, known as satellite cells, which represent the main pool of myogenic precursors responsible for post-hatch muscle growth and regeneration^6^.

Deviations from the optimal egg incubation temperature, particularly in terms of the timing of thermal manipulation, were shown to influence chicken phenotype at various developmental stages. Leg muscles exhibited distinct responses to early temperature manipulation (ED 4–7), with leg muscle weight decreasing at incubation temperatures of 38.5 °C^7^. Egg incubation temperature regimens above or below 37.5 °C showed altered intramuscular fat percentage following histochemical staining as well as a reduced muscle glycogen content in gastrocnemius muscle^7^. Positive effects of an egg incubation temperature variation were described on traits such as immune response, muscle and bone parameters, and productivity, hence being of potential practical relevance^8–10^. Moreover, disruptions from optimal conditions might alter mitochondrial function, with potential effects on skeletal muscle development and economic efficiency of poultry production^11,12^.

Higher egg incubation temperatures during the stages of embryogenesis (ED 0–5) were linked to higher myoblast proliferation rates^13^. Previous studies on chicken gene expression in breast muscle^14^ and in both breast and leg muscle^15^ indicated that variations in egg incubation temperature during ED 7–10 or 10–13 significantly influenced the embryonic transcriptome, associated with physiological and biochemical changes. Specifically, increased egg incubation temperature (38.8 °C) between ED 7–10 promoted the upregulation of genes involved in lipid metabolism, energy production, and cell maintenance in embryonic leg muscles. Similar effects were further confirmed by changes in the expression of heat shock proteins, growth factors, and muscle marker genes in response to egg incubation temperature variations^3,16,17^. Comparative gene expression studies of leg muscles from embryos at ED 10 and ED 13 further showed that changes in incubation temperature had an impact on muscle energy metabolism including mitochondrial respiratory activity^18^.

It is hypothesized that transient deviations from the standard egg incubation temperature during the second phase of embryonic myogenesis leave detectable transcriptomic signatures in leg muscle at hatch when assessed in day-old chicks. Due to contrasting selection of broiler and layer lines for muscle growth and egg production^19,20^, respectively, responsiveness to thermal shifts is expected to shape distinct transcriptomic profiles, reflecting the functional diversity in coping with environmental variations. The day-old state was therefore utilized as a proxy for the cumulative effects of the embryonic development. Accordingly, skeletomuscular transcriptomic responses are considered to exhibit divergent gene expression patterns and regulatory pathways in broilers and layers. Therefore, gene expression changes in the leg muscle of day-old Lohmann Brown (LB) layers and Ross 308 (Ross) broilers were profiled following a transient ED 10–13 thermal manipulation.

## Results

### Egg weight, hatching rate, hatch body weight and yolk-free body weight

**Fig. 1:**
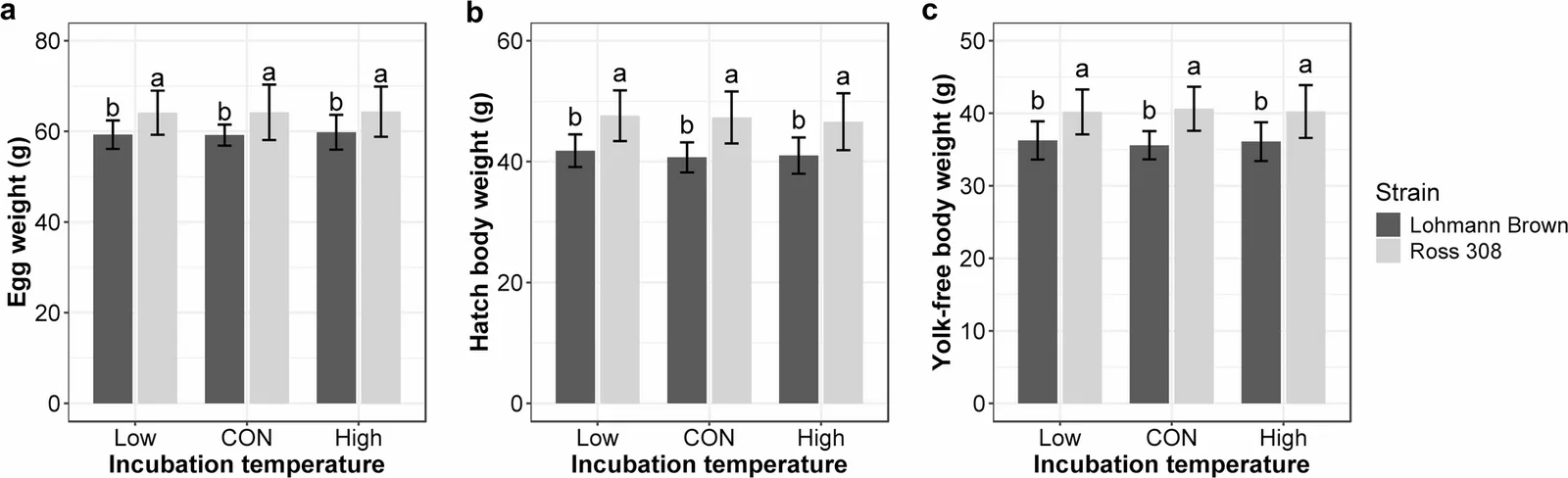
Egg weight (**a**), hatch body weight (**b**), and yolk-free body weight (**c**) across incubation temperature groups per strain. Lohmann Brown and Ross 308 eggs were incubated under standard conditions except during embryonic days 10–13, when variable incubation temperatures were applied (CON (37.8 °C), Low (36.8 °C), High (38.8 °C)). Data are presented as mean ± SD. ᵃᵇDifferent letters indicate significant differences (*P* ≤ 0.05).

Initial egg weight differed between the strains, with 59.4 ± 3.2 g for LB and 64.9 ± 5.4 g for Ross (mean ± SD; *P* < 0.001; Fig. 1a). Of 250 fertile and intact hatching eggs, 183 hatchlings were sampled (LB: 94; Ross: 89). The hatching rate was 76% for LB and 70% for Ross (*P* = 0.258). Within each strain, the Low and High groups exhibited unaltered hatching rates compared to their respective CON group (LB: *P* = 0.849; Ross: *P* = 0.154). The average hatching body weight (Fig. 1b) of LB was 41.2 ± 2.7 g (Low: 41.8 ± 2.7 g; CON: 40.7 ± 2.5 g; High: 41.0 ± 3.0 g) and of Ross 47.1 ± 4.4 g (Low: 47.6 ± 4.2 g; CON: 47.3 ± 4.3 g; High: 46.6 ± 4.7 g). Ross chicks had consistently higher body weights than LB chicks in all experimental conditions (*P* < 0.001). There were no significant differences in hatch body weights due to egg incubation temperature (*P* = 0.445) and sex (*P* = 0.075). A similar pattern was observed for yolk-free body weight (Fig. 1c). Ross chicks showed higher yolk-free body weights than LB chicks across all incubation temperature groups (*P* < 0.001), whereas incubation temperature (*P* = 0.771) and sex (*P* = 0.102) had no significant effects. Relative to the initial egg weight, hatch body weight represented 69.4 ± 3.3% in LB and 72.6 ± 2.5% in Ross (*P* < 0.001), while yolk-free body mass accounted for 87.4 ± 2.8% and 85.7 ± 3.7% of hatch body weight in LB and Ross, respectively (*P* < 0.001).

### Differentially expressed genes and their overlap

**Fig. 2:**
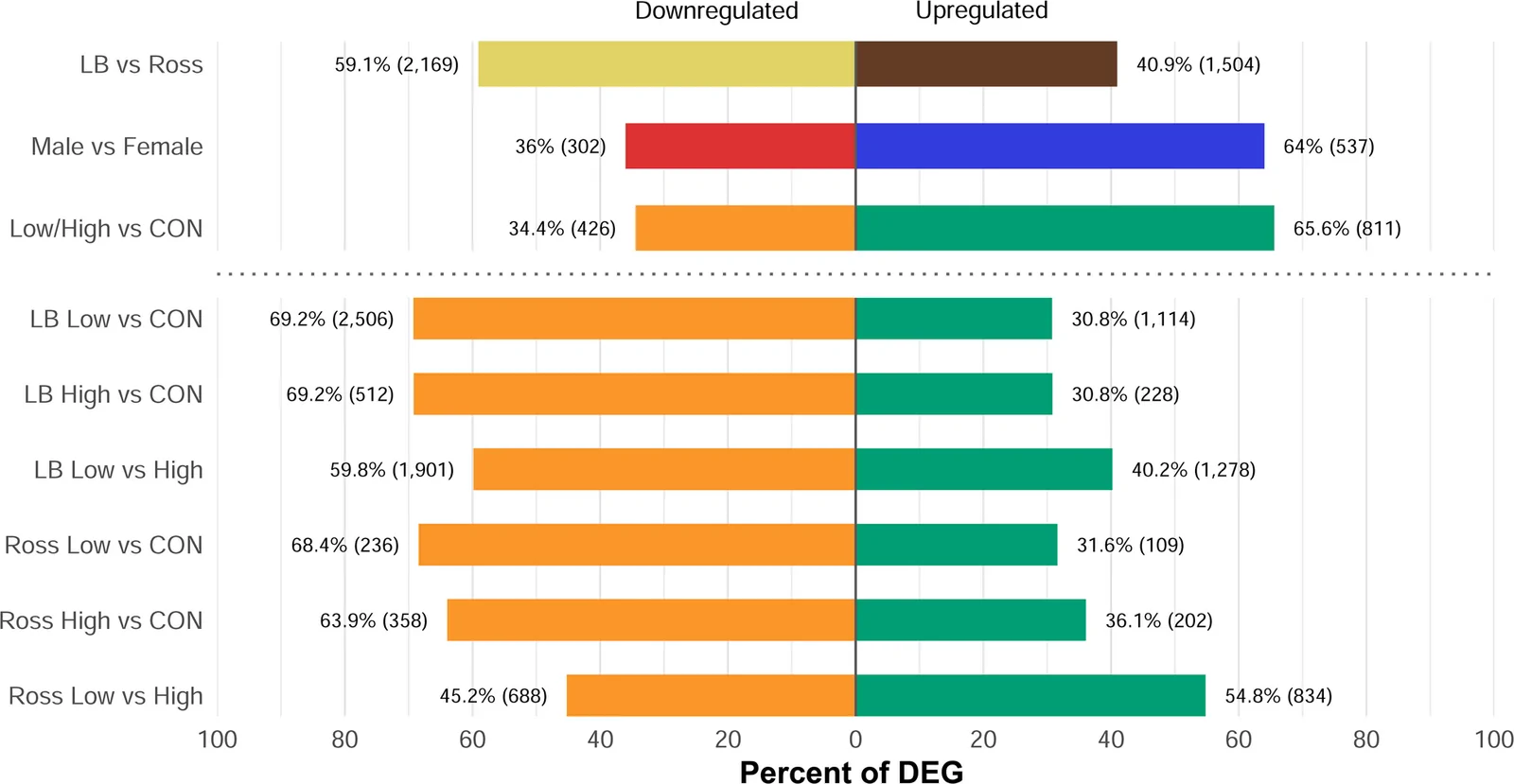
Differentially expressed genes (DEG) across the overall effects of strain, sex and incubation temperature variation (upper panel) as well as for hatched chicks of Lohmann Brown (LB) and Ross 308 (Ross) strains under different incubation temperature conditions at embryonic days (ED) 10–13 (*P* < 0.05; lower panel). Bars indicate the percentage of up-regulated (brown, blue, green) and down-regulated (yellow, red, orange) genes, with labels showing both percentages and absolute counts. For each contrast, the first experimental group specifies the reference direction used to classify DEG as up- or down-regulated.

**Fig. 3:**
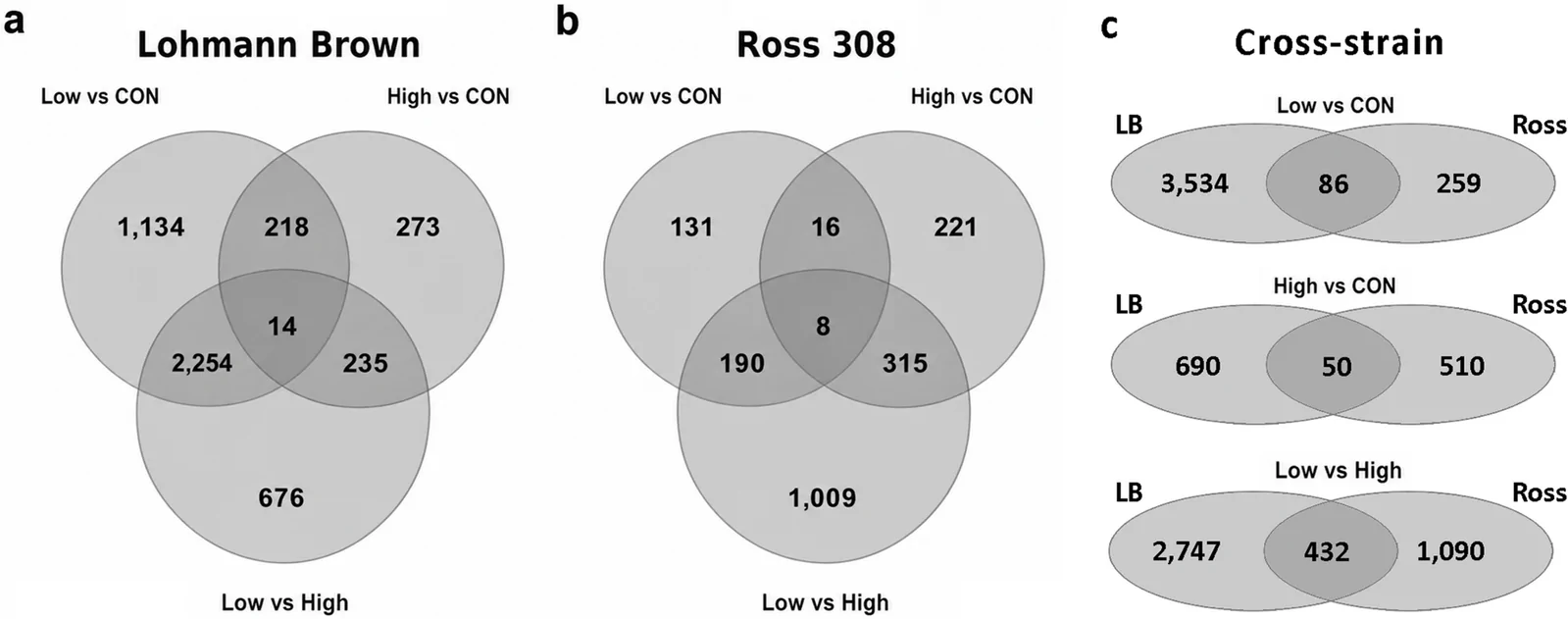
Intersections of differentially expressed genes (DEG) in (**a**) Lohmann Brown (LB), (**b**) Ross 308 (Ross), and (**c**) between strains following in-ovo thermal manipulation at embryonic days (ED) 10–13. The numbers represent nominally significant DEG (*P* < 0.05) in the respective contrasts between Low (36.8 °C), CON (37.8 °C) and High (38.8 °C) experimental groups.

Differential gene expression analysis was performed to compare gene expression patterns between strains (LB vs. Ross), sexes (female vs. male), and across the different incubation temperature conditions relative to the control (Low/High vs. CON) as shown in Fig. 2. Considering a nominal significance threshold of *P* < 0.05, a total of 3,673 DEG (adj. *P* < 0.05 with 2511 DEG) were identified for the strain difference (Supplementary information S1). The comparison between sexes resulted in 839 DEG (adj. *P* < 0.05 with 280 DEG), while 1237 DEG (adj. *P* < 0.05 with 95 DEG) were identified across incubation temperature variations relative to CON. Furthermore, DEG were obtained for LB and Ross strains across the three incubation temperatures at ED 10–13, accounting for the specific strain × temperature interaction. In the LB strain, 3620 DEG (Low vs. CON; adj. *P* < 0.05 with 2,561 DEG), 740 DEG (High vs. CON; adj. *P* < 0.05 with 1 DEG), and 3179 DEG (Low vs. High; adj. *P* < 0.05 with 1336 DEG) were identified at nominal significance threshold (Figs. 2 and 3). In the Ross strain, 345 DEG (adj. *P* < 0.05 with 0 DEG), 560 DEG (adj. *P* < 0.05 with 1 DEG), and 1522 DEG (adj. *P* < 0.05 with 50 DEG) were detected at *P* < 0.05 for Low vs. CON, High vs. CON, and Low vs. High, respectively. Overall, the Low vs. CON contrast showed the highest number of DEG in the LB strain, whereas in the Ross strain, the most pronounced number of DEG was observed in the Low vs. High contrast.

Analysis of DEG intersections within each strain revealed few genes common to all temperature contrasts (Fig. 3a and b; Supplementary information S1). The most divergent group, Low vs. High, overlapped substantially with both Low vs. CON and High vs. CON. While the Low vs. CON and High vs. CON comparisons in LB shared a considerable number of 232 DEGs, only 24 leg muscle genes were consistently altered by temperature variation relative to CON in the Ross strain. Furthermore, comparing DEG lists between strains across the three temperature contrasts (Fig. 3C) revealed low overlap, reaching 2.2%, 4.0%, and 10.1% in the Low vs. CON, High vs. CON, and Low vs. High contrasts, respectively.

### Functional enrichment analysis across main effects

**Fig. 4:**
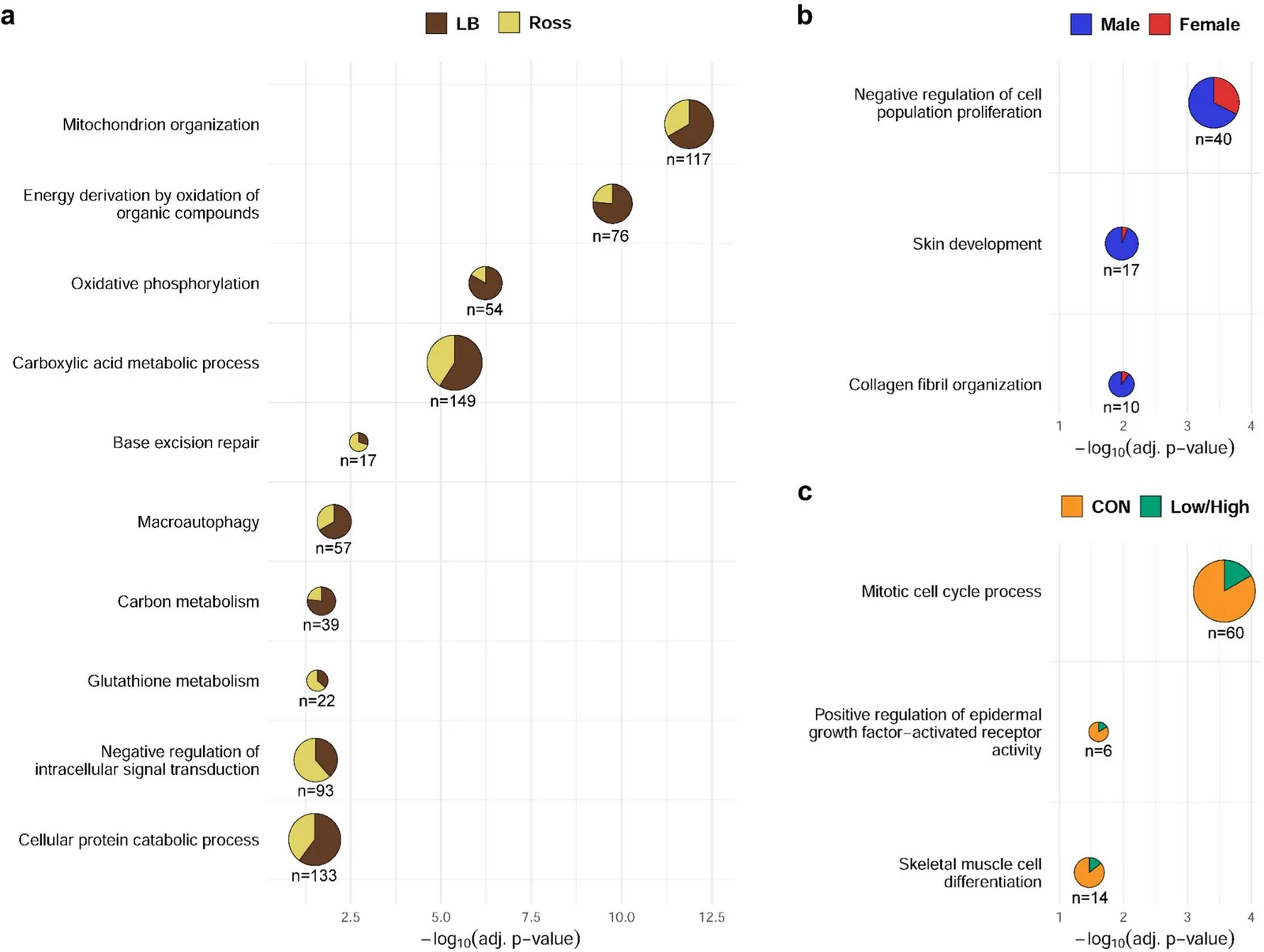
Enrichment of differentially expressed genes (DEG) in the main factors (**a**) chicken strain, (**b**) sex, and (**c**) variation of egg incubation temperature relative to CON birds. Significantly enriched GO terms for Biological Processes and KEGG pathways are represented, while similar terms are clustered by semantic similarity. The number of DEG representing the respective term is given below each pie chart. Colours show the share of up-regulated DEG in the respective experimental group within a contrast. The x-axis indicates enrichment significance as -log_10_(adjusted *P*-value).

Functional enrichment analysis of DEG for LB vs. Ross (input: 3673 DEG at nominal *P* < 0.05) revealed 17 GO:BP terms and 4 KEGG pathways indicating strain-specific differences in metabolic and cellular maintenance (Fig. 4; Supplementary information S1). Enrichment of mitochondrial and energy-related processes, including *mitochondrion organization* (GO:0007005), *oxidative phosphorylation* (KEGG:00190), *energy derivation by oxidation of organic compounds* (GO:0015980), and *carbon metabolism* (KEGG:01200), was characterized by the observed gene expression pattern in LB. In contrast, processes related to cellular resilience, including *glutathione metabolism* (KEGG:00480), *base excision repair* (KEGG:03410), and *negative regulation of intracellular signal transduction* (GO:1902532), were predominately represented by DEG with higher abundance in Ross compared to LB.

The analysis for sex-dependent enrichment (input: 839 DEG at nominal *P* < 0.05) showed the terms *negative regulation of cell population proliferation* (GO:0008285), *skin development* (GO:0043588), and *collagen fibril organization* (GO:0030199) enriched, comprising predominantly genes with higher abundance in the leg muscle of male chicks compared to females.

The overall evaluation of effects due to the egg incubation temperature (Low/High vs. CON; input: 1237 DEG at nominal *P* < 0.05) revealed divergent profiles of growth- and differentiation-related pathways (Fig. 4). Enrichment of the *mitotic cell cycle process* (GO:1903047) comprised predominatly genes with higher expression in the CON group compared to the transient incubation temperature variations. Similarly, *positive regulation of epidermal growth factor-activated receptor activity* (GO:0045742) and *skeletal muscle cell differentiation* (GO:0035914) were enriched, consisting primarily of genes with higher abundance in the CON group compared to those exposed to temperature variations.

### Functional enrichment analysis due to variable egg incubation temperature

**Fig. 5:**
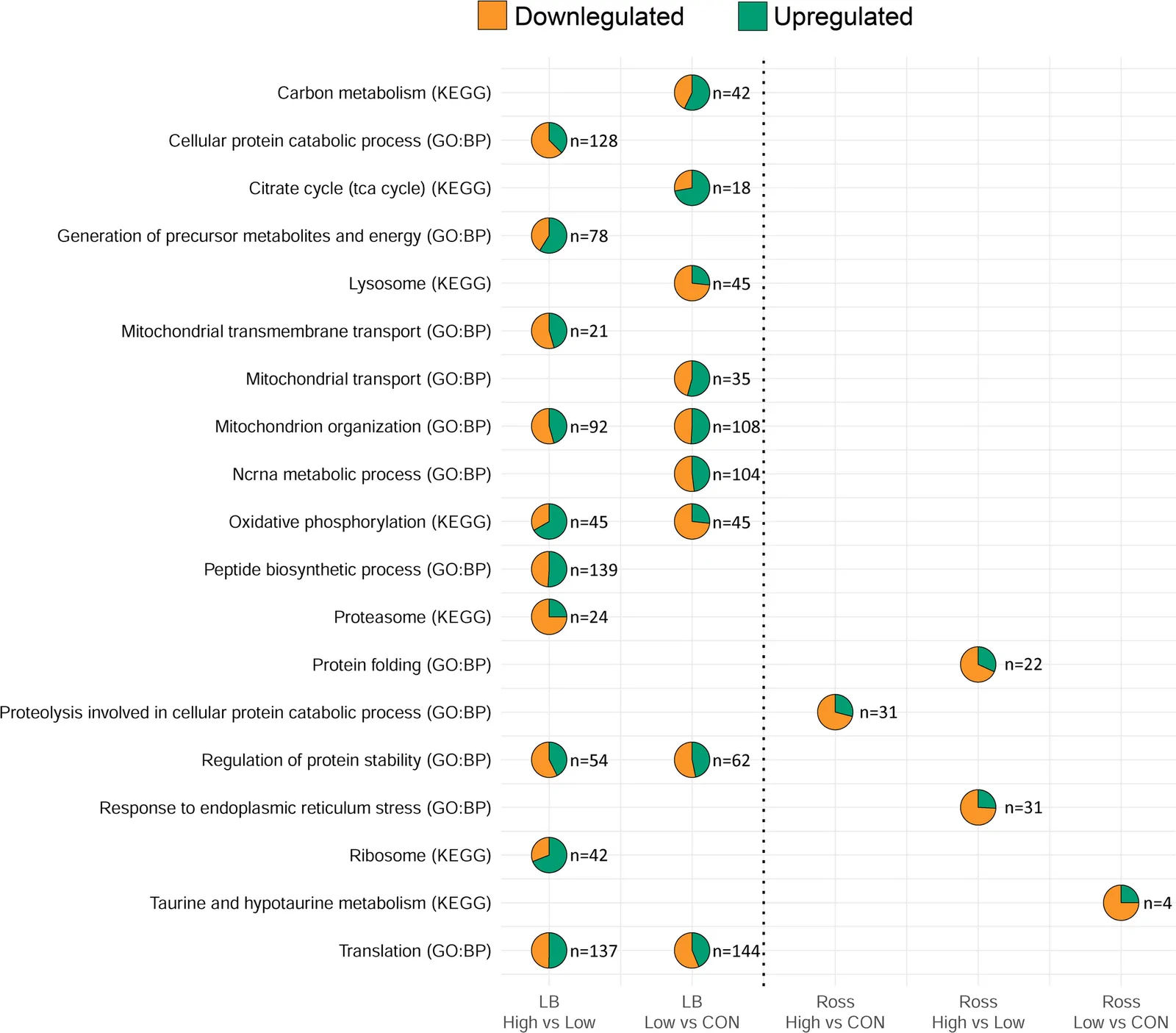
Functional enrichment of differentially expressed genes (DEG) in contrasts between Low (36.8 °C), CON (37.8 °C), and High (38.8 °C) egg incubation temperatures applied at embryonic days (ED) 10–13 in Lohmann Brown (LB) and Ross 308 (Ross) chicks. Significantly enriched GO:BP terms and KEGG pathways are shown, while similar terms are clustered by semantic similarity. The number of DEG representing the respective term is given next to each pie chart. The pie charts show the fractions of up-regulated (green) and down-regulated (orange) DEG contributing to each enrichment. Directionality of involved DEG is relative to the first group listed in each contrast stated as x-axis labels.

The LB Low vs. CON comparison (input: 3,620 DEG at nominal *P* < 0.05) displayed enrichment of processes related to mitochondrial function, protein synthesis and metabolic homeostasis (Fig. 5). Correspondingly, enriched GO biological processes included *mitochondrion organization* (GO:0007005), *translation* (GO:0006412), and *carbohydrate derivative biosynthetic process* (GO:1901137), respectively. Integration of enrichment results with DEG directionality indicates that DEG for *lysosome* (KEGG:04142) and *oxidative phosphorylation* (KEGG:00190) were predominantly lower abundant in the Low group compared to CON, whereas DEG assigned to *citrate cycle* (TCA cycle; KEGG:00020) were primarily up-regulated under lowered egg incubation temperature.

For the High vs. Low comparison in LB chicks (input: 3,179 DEG at nominal *P* < 0.05), enriched GO terms were primarily associated with mitochondrial function, ATP metabolism, and biosynthetic processes. Key enriched terms included *oxidative phosphorylation* (KEGG:00190), *ATP metabolic process* (GO:0046034), and *generation of precursor metabolites and energy* (GO:0006091). Processes related to protein biosynthesis in the leg muscle including *translation* (GO:0006412), *peptide biosynthetic process* (GO:0043043) as well as DEG related to *proteasomes* (KEGG:03050) and *ribosomes* (KEGG:03010) were also significantly enriched.

The LB High vs. CON comparison (input: 740 DEG at nominal *P* < 0.05) resulted in no significantly enriched terms.

In the Ross High vs. CON comparison (input: 560 DEG at nominal *P* < 0.05), enrichment signals included *proteolysis involved in cellular protein catabolic process* (GO:0051603). Among the DEGs annotated to this term, the predominant fraction was less abundant in High group compared to CON, indicating reduced transcription of several proteolysis/ubiquitin proteasome genes under high incubation temperature.

For the Ross High vs. Low contrast (input: 1522 DEG at nominal *P* < 0.05), enrichment was restricted to stress- and protein handling-related biological processes. Enriched GO terms included *protein folding* (GO:0006457) and *response to endoplasmic reticulum stress* (GO:0034976). Both terms are characterized by a predominance of down-regulated genes in the Ross High group compared with the Low group.

In the Ross Low vs. CON comparison (input: 345 DEG at nominal *P* < 0.05), enrichment analysis exclusively highlighted the *taurine and hypotaurine metabolism* (KEGG:00430) pathway.

### Target gene profiles

**Fig. 6:**
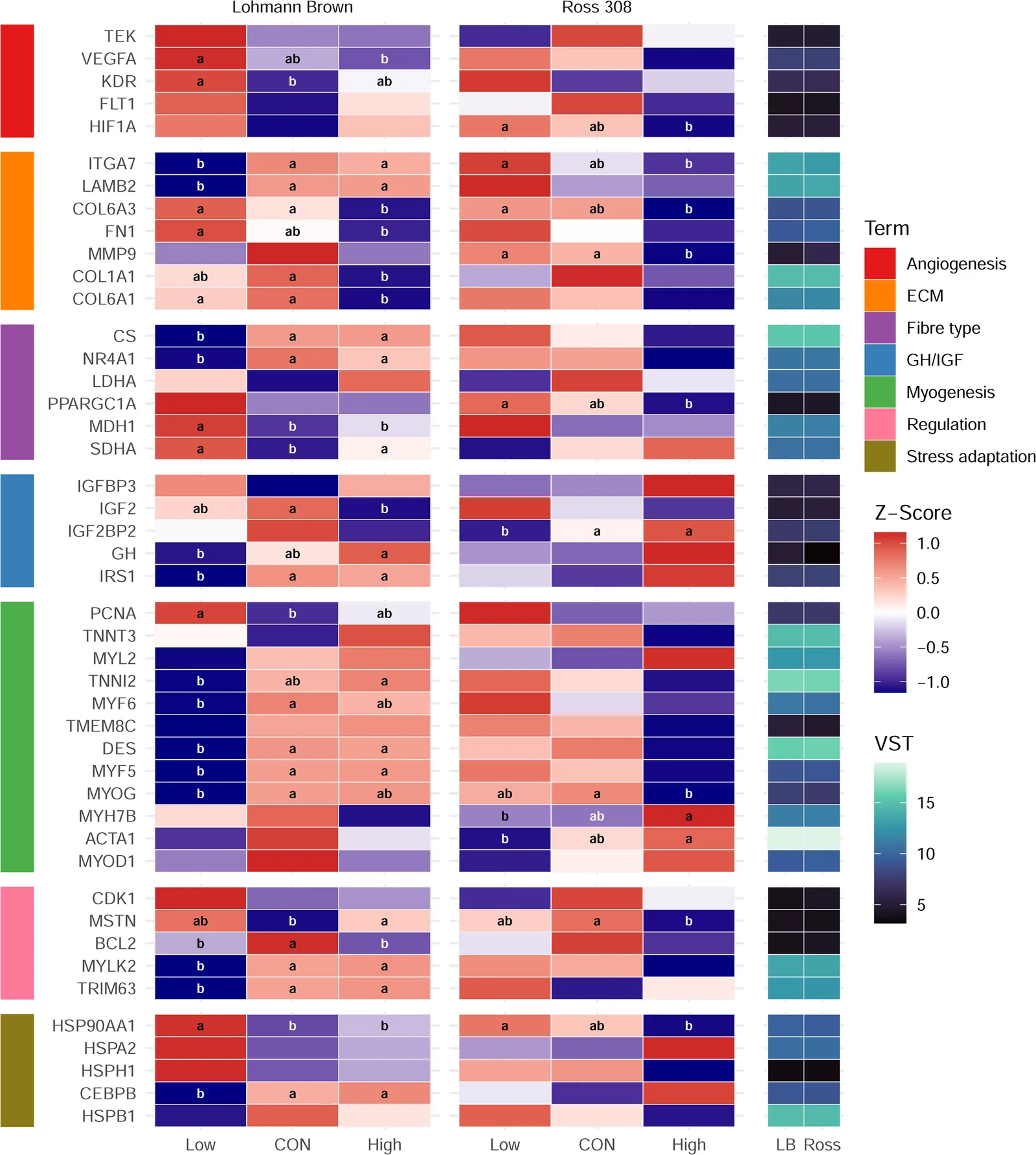
Differential expression of genes related to angiogenesis, extracellular matrix (ECM), fibre type, GH/IGF, myogenesis, cell cycle regulation, and stress adaptation in leg muscle at hatch dependent on variable egg incubation temperature applied at embryonic days (ED) 10–13 in Lohmann Brown (LB) and Ross 308 (Ross) chicks. The colours reflect scaled expression levels (Z-score calculated per row; blue-red) as well as mean variance stabilized expression levels (VST; blue-turquoise). ^a,b^Different letters indicate statistical difference between experimental groups separated for strain (*P* < 0.05). Respective gene names are displayed in Supplementary information S2.

The expression of genes grouped into the functional themes angiogenesis, extracellular matrix (ECM), fibre type, GH/IGF signaling, myogenesis, regulation, and stress was analysed to evaluate temperature-dependent expression patterns in LB and Ross chickens (Fig. 6). In the angiogenesis category, significant effects were observed for *VEGFA* and *KDR* in LB chicks. *VEGFA* was significantly higher expressed in the Low chicks compared to the High group. Moreover, the LB Low animals showed significantly higher *KDR* expression values compared to CON. In Ross, *HIF1A* displayed a significant treatment effect, with High differing from Low, while CON was not significantly different from either group.

In the ECM category, significant effects were observed for *ITGA7*, *LAMB2*, *COL1A1*, *COL6A1*, *COL6A3*, and *FN1* in LB. *ITGA7* and *LAMB2* were significantly lower expressed in the Low chicks compared to CON and High. The expression levels of *COL1A1*, *COL6A1*, *COL6A3*, and *FN1* were significantly lower in the High group compared to the Low group, CON group, or both. In Ross, significant treatment effects were observed for *ITGA7*, *COL6A3*, and *MMP9*. All three genes exhibited a significant downregulation in the High group compared to the Low group. Additionally, *MMP9* was also differentially expressed between High and CON in Ross.

In the fibre type theme, significant effects were observed for *CS*, *NR4A1*, *MDH1*, and *SDHA* in LB. *CS* and *NR4A1* were significantly lower expressed in the Low group compared to CON and High. *MDH1* was significantly higher expressed in the Low group compared to CON and High, whereas *SDHA* was up-regulated in High and Low compared to CON. In Ross, significant treatment effects were observed for *PPARGC1A*, with significant differences between High and Low.

In the GH and IGF system category, significant effects were observed for *IGF2*, *GH*, and *IRS1* in LB. *IGF2* was significantly down-regulated in High compared to CON, while *GH* was divergently expressed between High and Low. *IRS1* was expressed at significantly lower levels in Low compared to CON and High. In Ross, a significant treatment effect was observed for *IGF2BP2*, with significantly lower expression levels in Low compared to CON and High.

For genes related to myogenesis, significant effects were observed for *PCNA*, *TNNI2*, *MYF6*, *DES*, *MYF5*, and *MYOG* in LB. *PCNA* was significantly higher expressed in the Low group compared to CON, while the High group being not significantly different from either group. *TNNI2*, *DES*, *MYF5*, and *MYF6* revealed significantly lower expression levels in Low compared to CON and High. *MYOG* was significantly lower expressed in Low compared to CON. In Ross, significant treatment effects were observed for *MYOG*, *MYH7B*, and *ACTA1*. *MYOG* was significantly down-regulated in High compared to CON. *MYH7B* and *ACTA1* showed significantly higher expression levels in High compared to Low, whereas CON was not significantly different from either group.

In the theme comprising regulators of muscle development, significant effects were observed for *MSTN*, *BCL2*, *MYLK2*, and *TRIM63* in LB. *MSTN* was significantly higher expressed in the High group compared to CON. *BCL2* revealed a significant downregulation in the two thermal treatment groups compared to CON. The expression profiles of *MYLK2* and *TRIM63* showed a significantly lower abundance in Low compared to CON and High. In Ross, a significant treatment effect was observed for *MSTN*, which was significantly less abundant in High compared to CON.

In the category including cellular stress indicators, significant effects were observed for *HSP90AA1* and *CEBPB* in LB. *HSP90AA1* was significantly higher expressed in the Low group compared to CON and High. Vice versa, *CEBPB* expression was significantly down-regulated in Low compared to CON and High. In Ross, significant treatment effects were observed for *HSP90AA1*, which was significantly higher expressed in the Low group compared to High, while CON was not significantly different from either group.

## Discussion

### Strain-related differences

In the present study, clear differences between broiler (Ross) and layer (LB) chickens were observed at both phenotypic and transcriptomic levels at hatchi. As the hatching rate depends on factors such as genetics, hen age, and management^21^, the retrieved values reflected the experimental incubation protocol used in this study. Ross chicks exhibited higher body weight and yolk-free body mass at hatch than LB chicks, which is attributable to differences in initial egg weight between the strains. Clearly, egg size and associated metrics are not an independent covariate but integral component captured by the experimental factor strain. Similar observations have been reported previously, showing that broiler eggs contained larger absolute yolk reserves with higher metabolizable energy availability at hatch compared with layer eggs^22^. The observed differences reflect the contrasting breeding objectives of the two production types, i.e., broilers selected for rapid muscle growth and layers primarily selected for egg production. A pronounced genetic and physiological strain divergence was described accordingly^23,24^.

For RNA-seq data, the alignment rate was comparable between the two strains, indicating that sequence divergence of the broiler and layer strains from the reference genome did not introduce mapping bias. However, the transcriptomic analyses of leg muscle further supported strain-specific differences at hatch, culminating in pathways related to mitochondrial organization, oxidative phosphorylation, and carbon metabolism, which were characterized by a predominance of up-regulated genes in LB compared to Ross. Oxidative phosphorylation represents a central pathway for ATP production in skeletal muscle and contributes to metabolic specialization of muscle fibre types^25^. In mammals, myosin heavy chain isoforms are commonly used markers to distinguish muscle fibre types and reflect their metabolic properties^26^. It was demonstrated that layer chickens possess lower total myofibre numbers as well as fibre diameters in leg muscle compared to broiler chickens during post-hatch development^27,28^, with a lower proportion of fast glycolytic fibres^19^. Consequently, fast-growing broilers were shown to display distinct muscle fibre characteristics and higher glycolytic potential already at hatch^20^. These baseline differences in myofibre composition and metabolic profile provide a physiological context for the divergent transcriptomic responses with regard to oxidative phosphorylation and metabolism observed between broiler and layer chicks. Nevertheless, the gastrocnemius muscle accounts for a similar percentage of body mass in both broiler chickens and laying hens, representing approximately 1% of total body weight at 49 days of life^27^.

## Sex-related differences at hatch

With a total of 839 DEG, sex-specific differences in gene expression were observed to a lesser extent than differences between strains. Downstream analysis revealed the enrichment of ECM-associated pathways, which comprised primarily DEG with higher abundance in male chicks. Similar structural variation between sexes has also been reported in chicken thigh muscles, where males exhibited higher collagen content than females, suggesting differences in extracellular matrix organization and muscle architecture^29^. Moreover, previous RNA sequencing analyses at different post-hatch stages have identified numerous genes differentially expressed between male and female chickens in the skeletal muscles, many of which are involved in metabolic regulation and connective tissue development^30,31^. However, at hatch no significant differences were observed in body weight between male and female chicks in this and previous studies^32^, arguing for a mainly post-hatch development of the sexual dimorphism in chickens.

### Temperature sensitivity during egg incubation

In the present study, the transient egg incubation temperature manipulation during ED 10–13 did not significantly affect hatch body weight or yolk-free body mass in the two strains. Similar observations were previously reported in layer chickens, where elevated incubation temperature during late embryogenesis had no effect on chick body weight at hatch, although other physiological responses were observed^33^. However, deviations from the standard eggshell temperature were shown to alter embryonic growth patterns and chick viability depending on timing and duration of exposure^34^. Continuous exposure to elevated eggshell temperatures was shown to reduce skeletal development and hatchability^35^, while temperature variation close to hatching may increase embryonic mortality^36^. At the transcriptional level, comparison of leg muscle tissue from hatchlings incubated under altered temperature conditions (Low and High) with the CON group revealed enrichment of genes associated with the mitotic cell cycle process and skeletal muscle cell differentiation. This observation suggests that variation in incubation temperature at the critical stage of myofibre formation may affect cell proliferation and myogenic development of leg muscle as reported previously^15^.

### Consequences of a transient low egg incubation temperature

Incubation temperature reduction at ED 10–13 in LB enriched pathways associated with protein metabolism and mitochondrial function, including oxidative phosphorylation and the citrate cycle. The DEG enriched in the oxidative phosphorylation term, including *NDUFS2* and *NDUFS7* (complex I), *COX6A1* (complex IV), and *ATP5B* and *ATP5F1D* (complex V), were significantly down-regulated in the leg muscle under low incubation temperature conditions compared to CON (Supplementary information S1). The OXPHOS reduction/deficiency may have led to impaired ATP production and a disrupted ATP/ADP balance^37^. When considering mitochondrial function in the LB‑Low group in a broader context, an upregulation of several key genes of the tricarboxylic acid (TCA) cycle was observed, including *ACO1*, *ACO2*, *IDH1*, *IDH3A*, *MDH1*, and *SUCLA2*, whereas *CS*, *IDH2*, and *IDH3B* were down-regulated compared to High/CON animals (Supplementary information S1). This pattern points to a selective regulation of mitochondrial metabolic activity rather than a uniform shift. In particular, the downregulation of *CS* may reduce citrate formation from acetyl-CoA, which potentially limits citrate availability for lipid biosynthesis during avian embryonic development, consistent with chicken studies showing that citrate flux and export are tightly linked to lipogenesis^38^. Changes in TCA cycle-related metabolism were previously associated with altered muscle physiology and metabolic reprogramming in broiler chickens^39^. However, it is conceivable that the observed transcript pattern contributes to an anaplerotic replenishment of TCA cycle intermediates and thereby supports metabolic homeostasis during embryonic muscle development at low incubation temperatures^40^. Specifically, an increased abundance of *MDH1* may indicate an increased demand for redox equivalents (e.g., NADH), associated with a higher turnover of malate and oxaloacetate.

Most DEG associated with lysosomal degradation and recycling were significantly down-regulated in leg muscle cells of Low compared to CON LB chicks, which may suggest a reduced capacity for the turnover of cellular components as a result of the lower incubation temperature. Specifically, this pattern might be reflected by the downregulation of *LAMB2* and *ITGA7*, which may alter laminin organization and cell anchoring within the ECM as reviewed based on mammalian physiology^41,42^. Consequently, genes central to myogenesis and growth regulation (*MYF6*, *MYF5*, *MYOG*, *DES*, *GH*, *IRS1*) were down-regulated in Low chicks compared to CON chicks in the LB strain. Given the pivotal role of the IGF signaling system in regulating skeletal muscle growth and myogenesis in growing chickens,^43^. these findings collectively support the conclusion that lowering the incubation temperature at the critical developmental window during myofibre formation was associated with a transcriptomic profile consistent with suppressed muscle cell differentiation and altered leg muscle development in LB hatchlings. Whereas angiogenesis‑related processes showed increased expression of *VEGFA* and its receptor *KDR* in Low compared to High and CON chicks, respectively, this expression pattern indicates enhanced VEGF signaling. VEGFA is a central mediator of vascular development, promoting endothelial cell proliferation and capillary formation^44^, which likely reflects an adaptive increase in vascularization to sustain oxygen delivery to the developing muscle tissue. Indeed, a temperature-dependent oxygen regulation is known to play an important role in embryonic development, where hypoxia-responsive pathways help to maintain cellular oxygen balance^45^.

Notably, the Ross Low vs. CON comparison exhibited no overlapping results across the target functions of myogenesis, angiogenesis, and TCA cycle-related metabolism. These results indicate a distinct, strain-specific response to the temporary reduction in incubation temperature. The Ross hatchlings exhibited enrichment exclusively for taurine and hypotaurine metabolism under reduced incubation temperature compared to CON (Fig. 5). Taurine supports cellular homeostasis through its roles in antioxidant defence, osmoregulation, and mitochondrial function in metabolically active tissues^46^. The predominance of lower gene abundance in the Low group compared with CON for taurine and hypotaurine metabolism may reflect an increased vulnerability to oxidative stress and disrupted metabolic homeostasis during embryonic muscle development, potentially impacting muscle function and growth^47^.

### Consequences of a transient high egg incubation temperature

Following the higher incubation temperature at ED 10–13, several genes were found to be differentially expressed between High and CON in the two strains. However, analyses in LB hatchlings revealed that the transcriptional response was not coordinated at pathway level. In Ross hatchlings, expression profiles showed enrichment for proteolysis involved in cellular protein catabolic processes. Proteolysis, the enzymatic breakdown of proteins into amino acids, is a key component of cellular catabolism and plays a central role in regulating skeletal muscle protein turnover in growing chicks. Skeletal muscle size depends on the balance between protein synthesis and degradation, with excessive proteolysis leading to protein loss and altered tissue remodelling^48,49^. Here, genes showing lower abundance in the High group included critical core components of the Ubiquitin-Proteasome System, such as those involved in ubiquitin activation (*UBA6*), Cullin-based ligase scaffolding (*CUL1*, *CUL2*, *CUL4B*), and structural proteasome subunits (*PSMA2*, *PSMD14*), as well as ER-associated degradation adaptors (*SEL1L*, *DJC3*; Supplementary Table S1). This coordinated repression of core components indicates an overall suppression of intracellular protein catabolism and protein quality control pathways under high incubation temperature. Ultimately, this shift could promote greater net protein retention within muscle fibres during development, potentially contributing to differences in post-hatch muscle phenotype^50^.

No differential expression of key angiogenesis-related genes was detected at hatch between High and CON groups in either strain. This is consistent with reports suggesting that vascular responses might be impaired at higher incubation temperatures (i.e. ≥ 39 °C) or are positively influenced by hypoxic conditions^51,52^.

The expression of collagens such as *COL1A1*, *COL6A1*, *COL6A3* was reduced in High compared to CON chicks in leg muscle of LB chicks, indicating diminished ECM deposition during embryonic myogenesis. In the Ross High group, the decrease in *MMP9* expression relative to CON suggests a broader reduction in ECM remodelling, even in the absence of a significant reduction in collagen expression. Collagens are major structural components of the skeletal-muscle ECM and contribute to connective-tissue organization during myogenesis^53^, a process documented in differentiating embryonic chicken myoblasts^54^. Together, these profiles indicate an overall dampened ECM program, suggesting that a transient modification of incubation temperature altered the timing and regulation of myogenesis, with potential implications for post-hatch muscle development. In accordance, the lower *IGF2*, higher *MSTN*, and reduced *BCL* expression in LB chicks indicate a coordinated shift toward reduced myogenic growth and altered cell survival. In Ross chicks, similar alterations were suggested by the reduced expression of *MYOG* and *MSTN* in High compared with CON, although the lack of significance in other related genes indicates that this effect was selective rather than widespread. Together with the LB findings, these results point to a strain-dependent modulation of the timing and regulatory balance of embryonic muscle development.

## Conclusion

The transient ± 1 °C thermal deviations during ED 10–13 induced strain-dependent transcriptomic remodelling in leg muscle detectable at hatch. Distinct effects were observed under lowered incubation temperature including enrichment of mitochondrial pathways and downregulation of genes associated with myogenesis and growth signalling in LB but not in Ross hatchlings. Results suggest that lowering the incubation temperature at the critical developmental window during myofibre formation was associated with a transcriptomic profile consistent with suppressed muscle cell differentiation and altered leg muscle development in LB hatchlings. In contrast, a temporary increase in incubation temperature had a less pronounced effect in both LB and Ross, but still influenced proteolysis and resulted in an overall dampened ECM program. This suggests an altered timing and regulation of myogenesis in the developing leg muscle of chicks. The avian sexual dimorphism was not yet pronounced at hatch.

## Materials and methods

### Ethics, experimental design, and sampling

All experimental procedures were carried out at the Research Institute for Farm Animal Biology (FBN) in accordance with the German Animal Welfare Act and relevant institutional, national, and international guidelines and regulations. All experimental protocols were first reviewed by the Animal Care Committee of the Research Institute for Farm Animal Biology (FBN) and approved by the Landesamt für Landwirtschaft, Lebensmittelsicherheit und Fischerei Mecklenburg-Vorpommern under reference number LALLF 7221.3-1-022/24. This study is reported in accordance with the ARRIVE guidelines.

A total of 250 eggs were obtained from a commercial layer strain (Lohmann Brown; LB; Geflügelzucht Horstmann GmbH, Stolzenau, Germany) and a broiler strain (Ross 308; Ross; Artländer Geflügelhof, Quakenbrück, Germany). The experiment was conducted in five batches. Eggs were weighed before incubation and placed in the incubator under standard conditions, including controlled humidity and egg rotation until ED 10 (Grumbach Brutgeräte, Mücke, Germany). Environmental variables, specifically temperature and relative humidity, were consistently monitored throughout the incubation process using temperature and humidity dataloggers (EL-GFX-2+; Lascar Electronics, Whiteparish, United Kingdom). All eggs were screened on ED 10 and any unfertilised or cracked eggs were removed from the incubator. The remaining eggs were randomly distributed to experimental groups and three separate incubators (Grumbach Brutgeräte) based on three incubation temperatures applied between ED 10 and 13, i.e., control temperature (CON; 37.8 °C), higher temperature (High; 38.8 °C), or lower temperature (Low; 36.8 °C). After 72 h, all eggs were returned to standard incubation conditions at 37.8 °C. On ED 18, the eggs were transferred to a hatching incubator (Grumbach Brutgeräte), and incubated at 37 °C under standard conditions until hatch. On day 21 of incubation, the eggs were continuously monitored. Within 24 h after hatching, 183 chicks were sampled. For the five batches, the numbers of hatched eggs relative to fertilised and intact eggs were (i) *n* = 28/44, (ii) *n* = 37/55, (iii) *n* = 40/49, (iv) *n* = 54/72, and (v) *n* = 24/30, respectively. The body weight was recorded prior to slaughter. The chicks were stunned and euthanized by decapitation, after which the yolk sac was dissected and weighed. The leg muscle samples including the gastrocnemius muscle were collected, snap-frozen in liquid nitrogen, and stored at -80 °C until analysis. Sex was determined post mortem by PCR^55^.

### RNA extraction, library preparation, and sequencing

From 183 sampled chicks, a subset of 108 individuals from four batches was selected for RNA sequencing based on RNA quality and to ensure a balanced representation of strain, incubation temperature, sex, and batch. The 108 chicks comprised *n* = 18 (9f/9m) for LB-CON, *n* = 18 (9f/9m) for LB-Low, *n* = 18 (10f/8m) for LB-High, and *n* = 20 (9f/11m) for Ross-CON, *n* = 16 (10f/6m) for Ross-Low, *n* = 18 (9f/9m) for Ross-High, which sums up to *n* = 56 female chicks and *n* = 52 male chicks. Detailed sample information including strain, sex, incubation temperature, and sampling batch is provided in Supplementary information S3. Each 30–50 mg per sample of frozen leg muscle was used for QIAzol-based RNA extraction followed by a clean-up with the RNeasy Mini Kit (Qiagen, Hilden, Germany). The procedure followed the manufacturer’s guidelines and included an initial bead-based disruption of the tissue using the Precellys 24 homogenizer (PEQLab Biotechnology GmbH, Darmstadt, Germany). In addition, an on-column DNase digestion step was performed. RNA concentration and purity were assessed using NanoDrop 2000, and RNA integrity was evaluated using the Agilent Bioanalyzer (Agilent Technologies Inc., California, USA). The RNA integrity number (RIN) had an average of 8.6 ± 0.27 (mean ± SD). Total RNA was used for mRNA sequencing library preparation using the Illumina Stranded mRNA library preparation kit according to the manufacturer’s protocol (Illumina, San Diego, CA, USA). The DNA libraries were quality checked using a DNA 1000 chip (Agilent Technologies) and quantified for molar concentration using the Qubit dsDNA HS assay kit (Invitrogen, Darmstadt, Germany). Sequencing was performed on the NextSeq 2000 (Illumina, San Diego, CA, USA) at the sequencing facility of the Research Institute for Farm Animal Biology (FBN), Dummerstorf, Germany.

### Pre-processing of sequencing reads

Raw sequencing reads were collected from 108 leg muscle samples and converted to FASTQ files from the base call (BCL) file format using BCL Convert (v4.2.7). Adapter-like sequences were removed using Trim Galore (v0.6.10). The reads were then subjected to FastQC (v0.11.9) for quality control, and the raw reads were pre-processed by removing low-quality reads with a mean Q-score of less than 20 and short reads of less than 20 base pairs. The reads that passed quality control were considered for downstream analysis. HISAT2 (v2.2.1), an alignment program for mapping high-throughput next-generation sequencing reads, was used in the nf-core/ rna-seq pipeline with default parameters^56^. Reads were mapped to the chicken reference genome GRCg6a (Ensembl release 114). Read counts were assigned for features using htseq-count with the default ‘union’ mode (v2.0.2) as defined by the Ensembl GTF file^57^. The RNA sequencing of broiler and layer leg muscle yielded an average of 39.51 million total reads per sample, of which 80.2% on average were aligned to the chicken reference genome GRCg6a (LB: 79.9 ± 4.9%; Ross: 80.5 ± 4.9%).

### Statistics, transcriptomics and pathway analysis

Phenotypic data were analysed in R (R Core Team, v4.4.1). Hatching rates per strain as well as per incubation temperature group within each strain were tested for significance using the chi-square test. The effects of strain, incubation temperature, sex, and sampling batch on egg weight, hatch body weight, and yolk-free body weight as well as the associated ratios (i.e., hatch body weight relative to initial egg weight; yolk-free body weight relative to hatch body weight) were tested using analysis of variance (ANOVA). Post-hoc comparisons were performed using Tukey’s HSD. *P*-values < 0.05 were considered statistically significant.

The read count data of RNA sequencing were assessed by using the arrayQualityMetrics package (v3.56.0) to check sample-to-sample distances, distributions of counts, and differences between the log2 counts of a gene in one sample and the corresponding log2 average across all samples^58^. Based on these metrics, all samples were kept for further processing. Differential gene expression analysis was carried out using the Wald test implemented in the DESeq2 R package^59^. The statistical model included the combined group factor (strain × incubation temperature), sex, and sampling batch. Main effects for strain, sex, and incubation temperature were calculated by defining linear contrasts that averaged the coefficients of the combined group factor. For instance, the main effect of temperature was derived by comparing the mean of all high and low-temperature groups against the mean of all CON temperature groups, accounting for strain and sex. Moreover, differentially expressed genes (DEG) for strain-specific temperature effects were retrieved in the contrasts LB Low vs. CON, LB High vs. CON, LB High vs. Low, Ross Low vs. CON, Ross High vs. CON, and Ross High vs. Low. To enhance the statistical power for identifying DEG, pre-filtering to retain only data from genes with 20 or more raw counts in at least 12 samples was performed. Genes were reported when meeting either the nominal significant threshold *P*-value < 0.05 or the Benjamini-Hochberg adjusted *P*-value < 0.05 as indicated.

Functional enrichment analyses of Gene Ontology: Biological Processes (GO:BP) terms and Kyoto Encyclopedia of Genes and Genomes (KEGG) pathways^60^ were conducted using g:Profiler (version e106_eg53_p16_ef2524f6). To ensure a sufficient number of genes for robust enrichment analysis, DEG identified considering a nominal significance threshold (*P* < 0.05) were incorporated. This approach captures the small-effect transcriptional changes induced by transient temperature variation, while the risk of false positives is mitigated by applying multiple testing corrections at the pathway level. Accordingly, terms with an adjusted *P*-value < 0.05 were considered significantly enriched. Enriched terms were filtered by term size (≤ 500 genes) to exclude generic descriptive terms. To further reduce redundancy for visualization, GO:BP terms were clustered using Wang’s semantic similarity implemented in the simplifyEnrichment R package (v2.4.1). The KEGG pathways were summarized using a Jaccard overlap threshold to indicate term name similarity of 0.5.

The RNA-seq data were further investigated for the expression of specific candidate genes. The panel comprised genes involved in angiogenesis (*FLT1*, *HIF1A*, *KDR*, *TEK*, *VEGFA*), extracellular matrix (ECM) structure and remodelling (*COL1A1*, *COL6A1*, *COL6A3*, *FN1*, *ITGA7*, *LAMB2*, *MMP9*), muscle fibre type and oxidative metabolism (*CS*, *LDHA*, *MDH1*, *NR4A1*, *PPARGC1A*, *SDHA*), the growth hormone (GH)-insulin-like growth factor (IGF) axis (*GH*, *IGF2*, *IGF2BP2*, *IGFBP3*, *IRS1*), myogenic determination and differentiation (*ACTA1*, *DES*, *MYF5*, *MYF6*, *MYH7B*, *MYL2*, *MYOD1*, *MYOG*, *PCNA*, *TMEM8C*, *TNNI2*, *TNNT3*), regulatory factors (*BCL2*, *CDK1*, *MSTN*, *MYLK2*, *TRIM63*), and genes associated with cellular stress adaptation and heat shock response (*CEBPB*, *HSP90AA1*, *HSPA2*, *HSPB1*, *HSPH1*). Respective gene names are listed in Supplemental information S2. The selection of genes and key processes was derived from previous studies on skeletal muscle development and growth in chickens^61–63^. Results were visualized in a heatmap using ggplot2 in R (v4.0.0).

## Supplementary Information

Below is the link to the electronic supplementary material.


Supplementary Material 1



Supplementary Material 2



Supplementary Material 3


## Data Availability

Data are available from the EMBL-EBI database (www.ebi.ac.uk/arrayexpress) under the accession number E-MTAB-16972.
